# Heme Degrading Protein HemS Is Involved in Oxidative Stress Response of *Bartonella henselae*

**DOI:** 10.1371/journal.pone.0037630

**Published:** 2012-05-31

**Authors:** MaFeng Liu, Henri-Jean Boulouis, Francis Biville

**Affiliations:** 1 Université Paris-Est, Ecole nationale vétérinaire d'Alfort, UMR BIPAR INRA-Anses-UPEC-ENVA, Maisons-Alfort, France; 2 Key Laboratory of Zoonosis, Ministry of Education, Institute of Zoonosis, Jilin University, Changchun, People's Republic of China; 3 Département de Microbiologie, Pasteur Institute, Paris, France; East Carolina University School of Medicine, United States of America

## Abstract

*Bartonellae* are hemotropic bacteria, agents of emerging zoonoses. These bacteria are heme auxotroph *Alphaproteobacteria* which must import heme for supporting their growth, as they cannot synthesize it. Therefore, *Bartonella* genome encodes for a complete heme uptake system allowing the transportation of this compound across the outer membrane, the periplasm and the inner membranes. Heme has been proposed to be used as an iron source for *Bartonella* since these bacteria do not synthesize a complete system required for iron Fe^3+^uptake. Similarly to other bacteria which use heme as an iron source, *Bartonellae* must transport this compound into the cytoplasm and degrade it to allow the release of iron from the tetrapyrrole ring. For *Bartonella*, the gene cluster devoted to the synthesis of the complete heme uptake system also contains a gene encoding for a polypeptide that shares homologies with heme trafficking or degrading enzymes. Using complementation of an *E. coli* mutant strain impaired in heme degradation, we demonstrated that HemS from *Bartonella henselae* expressed in *E. coli* allows the release of iron from heme. Purified HemS from *B. henselae* binds heme and can degrade it in the presence of a suitable electron donor, ascorbate or NADPH-cytochrome P450 reductase. Knocking down the expression of HemS in *B. henselae* reduces its ability to face H_2_O_2_ induced oxidative stress.

## Introduction

*Bartonella* species are now well established as human pathogens responsible for several emerging zoonoses [1]. *Bartonella bacilliformis* (*B. bacilliformis*), *Bartonella quintana* (*B. quintana*) and *Bartonella henselae* (*B. henselae*) are the most medically important species although several others have also been described as pathogens [2]. *B. henselae* is now recognized as one of the most common zoonoses acquired from companion animals in industrialised countries [3]. The bacterium causes cat scratch disease as well as being increasingly associated with a number of other syndromes [4]. Most *Bartonella* species appear to share a similar natural cycle that involves arthropod transmission, then exploitation of a mammalian host. Each *Bartonella* species appears to be highly adapted to one or few reservoir hosts in which *Bartonella* causes a long lasting intra-erythrocytic bacteraemia as a hallmark of infection. The bacterial persistence in erythrocyte is an original strategy for its persistence in its host and the resulting long-lasting intraerythrocytic bacteraemia is considered to represent a unique adaptation to the mode of transmission by blood sucking arthropod vectors. Flagella [5], a deformin activity [6], and a locus containing *ialA* and *ialB* genes [7] were shown to be important for erythrocytes invasion by *B. bacilliformis*. Also, the importance of the Trw T4SS for erythrocytes invasion was demonstrated for *B. tribocorum* [8] and *B. birtlesii* [9]. The erythrocytes invasion can also be proposed to be a strategy for *Bartonella* species to get heme that is absolutely required for the growth of *B. henselae*. It was also shown that addition of heme in an iron depleted medium supported the growth of *B. henselae*, thus showing the use of heme as an iron source [10]. Analysis of the complete genomic sequences of *B. quintana* and *B. henselae* supports the absolute requirement for heme since these two *Bartonella* species do not contain genes encoding for heme biosynthesis [11]. All the sequenced *Bartonella* genomes indicate that all these bacteria do not encode for siderophore biosynthesis and a complete iron Fe^3+^ transport system. Only genes sharing strong homologies with all the compounds of a Fe^2+^ uptake system already characterized in *Yersisna pestis* [12], and *Photorhabdus luminescens* [13] are present in *Bartonella* genomes. *Bartonella* genomes encode for a complete heme transport system that was shown to be active in the presence of high heme concentration for *B. quintana* [14]. In the cluster of genes devoted to heme uptake, one gene, *hemS*, shares homology both with heme degrading enzymes and heme trafficking enzymes. The activity of heme degrading enzymes is required for the release of iron from heme after its transportation into the cytoplasm [15]. Different activities allowing the release of iron from heme have been characterized already. The more common, is a heme oxygenase first put in evidence in *Corynebacterium diphtheria* [16]. Other bacterial heme oxygenases, that provoke the release of iron by degrading heme to billiverdin were also characterized in Gram negative and Gram positive bacteria such as *Pseudomonas aeruginosa* [17], or *Clostridium tetani* [18] (for a review see: [15]). Other heme degrading mono-oxygenases were identified in Gram positive bacteria such as *Bacillus anthracis* [19] and Gram negative bacteria such as *Bradyrhyzobium japonicum* [20]. Protein sequestering heme have been identified in bacteria such as *Yersinia enterocolitica* [21], *Shigella dysenteriae* [22], *Pseudomonas aeruginosa* [23]. Enzyme allowing the release of iron from protoporphyrin IX leaving ring intact was identified in *Escherichia coli* (*E. coli*) [24]. Controversial data about this heme dechelatase activity were published recently [25].In this report, we investigated the function of HemS for *B. henselae*, using functional complementation, and partial biochemical characterization. The effect of *hemS* knock down was also investigated in *B. henselae*.

## Materials and Methods

### Bacterial strains and plasmid

Bacterial strains and plasmids used in this study are listed in Table 1.

**Table 1:** Strains and plasmids used in this study.

*E. coli* strains	Genotype	Source or reference
XL1-blue	F^−^ *supE44 hdsR17 recA1 endA1 gyrA46 thi relA1* lac^−^ F′ *proAB^−^* l*acI^q^ lacZΔM15 Tn10 (Tet^R^)*	Laboratory collection
JP313	*araD139 relA rpsL 150 thi flb5301 (lacU 139*) *deo7 ptsF25* Δ*ara 174*	Laboratory collection
MG1655 *ttdA::Cmp*	F^−^ *ttdA::Cmp* Cmp^R^	[31]
FB8.27	F^−^, *Δlac* X74, *entF::TnphoA′5*, Tet^R^	[37]
FB8.27 (pAM238*::hasR*)	FB8.27 pAM238*::hasR*, Tet^R^, Spc^R^	[24]
FB8.27 *efeB::Kan yfeX::Cmp* (pAM238*::hasR*)	FB8.27 *efeB::Kan yfeX::Cmp*, pAM*::hasR*, Tet^R^, Spc^R^, Kan^R^, Cmp^R^	[24]
XL1-blue (pBAD24:: *hemS_his_*)	XL1-blue, pBAD24::*hemS*_his_, Amp^R^	This study
JP313 (pBAD24)	JP313 pBAD24 Amp^R^	This study
JP313 (pBAD24*::hemS_his_*)	JP313 pBAD24::*hemS*_his_ Amp^R^	This study
FB8.27(pAM238*::hasR*), (pBAD24)	FB8.27 pAM238*::hasR,* pBAD24, Tet^R^, Spc^R^, Amp^R^	This study
FB8.27 *efeB::Kan yfeX::Cmp* (pAM238*::hasR*), (pBAD24)	FB8.27 *efeB::Kan yfeX*, pAM*::hasR*, pBAD24, Tet^R^, Spc^R^, Kan^R^, Amp^R^, Cmp^R^	This study
FB8.27 *efeB::Kan yfeX::Cmp* (pAM238*::hasR*), (pBAD24::*hemS*_his_)	FB8.27 *efeB::Kan yfeX::Cmp*, pAM*::hasR*, pBAD24::*hemS*_his_, Tet^R^, Spc^R^, Kan^R^, Amp^R^, Cmp^R^	This study

*Bartonella* strains	Genotype	Source or reference
*B. henselae*	*B. henselae* Houston-1, ATCC 49882^T^	Laboratory collection
*B. henselae* (pNS2Tr)	*B. henselae* Houston-1(pNS2Trc) Kan^R^	This study
*B. henselae* (pNS2Trc*::hemS*_AS_)	*B. henselae* Houston-1, pNS2Trc*::hemS*_AS_, Kan^R^	This study

Plasmids		
pKOBEGA	λred functions, Amp^R^	[49]
pBAD24	pBR322 araC, arabinose-inducible promoter, Amp^R^	[50]
pNS2Trc	Kan^R^	[41]
pAM238*::hasR*	pAM238*::hasR* Spc^R^	[51]
pBAD24::*hemS*_his_	pBAD24 carrying *B. henselae hemS_his_*, Amp^R^	This study
pNS2Trc*::hemS*_AS_	pNS2Trc carrying antisense *hemS* from *B. henselae*.	This study

### Media and growth conditions

Bovine hemoglobin (Hb), and 2, 2′dipyridyl (Dip) were obtained from Sigma Chemical. Heme was dissolved immediately before use in 0.02 M NaOH. Hb was dissolved in 100 mM NaCl. Heme and Hb solutions were filter-sterilized with 0.26 µm pore size Millipore filters for bacterial growth experiments. *E. coli* strains were grown on LB medium or M63 minimal medium aerobically at 37°C [26]. M63 medium was supplemented with 0.4% glycerol (Gly) as carbon source. Solid media contained 1.5% Difco agar. Soft M63 agar medium contained 0.7% Difco agar. Iron-depleted medium was obtained with the addition of Dip at a 70 µM final concentrations. Antibiotics were added to the following final concentrations (µg ml^−1^): Ampicillin (Amp), 50; Kanamycin (Km), 50; Spectinomycin (Spc), 50, Chloramphenicol (Cmp), 20. Arabinose was added at 0.2% for induction of the P_ara_ promoter as indicated. *B. henselae* was grown on Columbia blood agar (CBA) plate containing 5% defibrinated sheep blood (Biomérieux; ref 43041), or in Schneider's medium (Gibco) supplemented with 10% fetal calf serum [27] at 35°C under 5%CO_2_ atmosphere.

### Use of heme as iron source: *E. coli* assay

Tested strains were grown at 37°C for 18 hours in M63 medium without iron, with 0.4% glycerol as carbon source, and in the presence of 0.2% arabinose. Culture were checked for OD at 600_nm_ and a 100 µl sample of an overnight culture of tested strain adjusted to OD_600_ = 1 was mixed with 4 ml of soft agar and poured onto M63 plates containing 0.4% Glycerol, 0.2% arabinose, and 70 µM Dip (M63D). Wells (5 mm in diameter) were cut in the agar and filled with 100 µl of 50 µM, 10 µM, 5 µM, or 1 µM of filter sterilized Hb solution. Growth around the wells was recorded after one and two day incubation at 37°C. All experiments were performed in triplicate.

### Effect of the *hemS* knockdown in *B. henselae*

To evaluate the effect of *hemS* knock down on growth of *B. henselae*, tested strains were grown both in Schneider's liquid medium and on CBA plates. *B. henselae* (pNS2Trc) and *B. henselae* (pNS2Trc*::hemS*_AS_) were collected after 5 days growth on CBA plates and suspended in Schneider's medium. The OD_600_ of the bacterial suspension was adjusted to 0.05. Two ml of this suspension were poured into 12 wells plate and grown at 35°C in the presence of 5% CO_2_. The OD_600_ was checked at day 2, 4, 6, and 7 after inoculation. Serial dilutions of bacterial suspension were plated on CBA plates, and the colony size was evaluated after 6 and 10 days of growth at 35°C in the presence of 5% CO_2_.

### H_2_O_2_ challenge

*B. henselae* (pNS2Trc) and *B. henselae* (pNS2Trc*::hemS*_AS_) were grown on CBA plates during 5 days at 35°C under 5% CO_2_ atmosphere. Bacteria collected from one plate were suspended, and washed twice in PBS buffer. Cell suspension was then diluted to OD_600_ 0.5. Before H_2_O_2_ challenges, serial dilutions of the tested cell suspension were spread on CBA plates (T_0_). For challenge assay, bacteria were incubated 30 minutes in PBS buffer in the presence of 1 mM, or 10 mM H_2_O_2_ at 35°C under 5%CO_2_ atmosphere. After exposure to H_2_O_2_, bacteria were washed twice in PBS buffer and several dilutions plated on CBA plates (T_1_). After 15 days incubation at 35°C under 5% CO_2_ atmosphere, colonies were counted. Survival rate was expressed by (T_1_/T_0_)×100%. All experiments were performed in triplicate.

### Genetic techniques

*E. coli* cells were transformed by the calcium chloride method [28]. P1 lysates and transductions were performed as previously described by Miller [26]. *Bartonella* cells were transformed by electroporation as previously described [29].

### Nonpolar deletion of *yfeX* in *E. coli* by red linear DNA gene inactivation

A non polar mutation that deletes the entire *yfeX* gene was created by allelic exchange as previously described [30]. Briefly, plasmid pKOBEGA (an ampicillin-resistant derivative of pKOBEG) (see Table 1) was introduced into the target strain, and electrocompetent cells were prepared at 30°C after induction of the λ red system carried by pKOBEGA with 0.2% arabinose. A three-step PCR procedure was used to produce a PCR product in which the *cat* gene from pHP45Ω (9) is flanked by 500-bp homology arms corresponding to DNA regions located upstream and downstream from the *yfeX* start and stop codons, respectively. The following primers were used: for the left 500-bp *yfeX* homology arm, AmtAmtyfeX and AmtAvlyfeX and for the right 500-bp *yfeX* homology arm, AvlAmtyfeX and AvlAvlyfeX. The *cat* gene cassette (0.9 kb) was amplified from strain *E. coli* MG1655 *ttdA*::Cm [31] using the primers Cat5 and Cat3. The PCR product resulting from the three-step procedure was introduced into *E. coli* XL1 blue/pKOBEGA using electroporation, and chloramphenicol-resistant deletion mutants produced by allelic exchange were selected at 37°C (to eliminate the thermosensitive plasmid pKOBEGA). Correct chromosomal insertion was checked by PCR amplification using the cat primers Cat5 and Cat3 in combination with AvlAvlyfeX and AmtAmtyfeX, respectively. The *yfeX::Cmp* mutation was then introduced in strain FB8.27 *efeB::Kan* using P1 transduction.

### DNA manipulations

*B. henselae* chromosomal DNA was isolated using the Wizard Genomic DNA purification kit (Promega). Small-scale plasmid DNA preparation was performed by using a QIAprep Spin Miniprep kit (Qiagen). Restriction endonuclease digestions, and ligation were carried out according to the manufacture's recommendation. DNA fragments were amplified in a Hybaid PCR thermocycler using Phusion DNA polymerase (Finnzymes). Nucleotide sequencing was performed by Eurofins MWG Operon. Purification of DNA fragments from PCR reaction, restriction reaction, and agarose gels were performed using Macherey-Nagel NucleoSpin® Extract II kit.

### Construction of a recombinant vector expressing HemS from *B. henselae*

The entire coding region of *hemS* was amplified by PCR from the *B. henselae* chromosomal DNA using primers hemShamont, containing NheI restriction site and allowing addition of a His_6_ –tag at the N-terminus part of the protein, and primer hemShaval containing a KpnI restriction site (Table 2). The 1070 bp PCR product was purified, digested by NheI and KpnI, and ligated with plasmid pBAD24, digested with NheI and KpnI, to give plasmid pBAD24::*hemS*_his_. Ligation product was introduced in *E. coli* strain XL1 blue, using calcium chloride method. Transformants were screened using PCR method with hemShamont and hemShaval primers. Six PCR positive clones were then sequenced.

**Table 2:** Primers used in this study.

Primer	Gene	Organism	Sequence
hemShamont	*hemS*	*B. henselae*	5′TTTTGGGCTAGCAGGAGGAATTCACCATGCATCATCACCATCACCATTCATATACAGCCGAAAT 3′
hemShaval	*hemS*	*B. henselae*	5′ATCCCCGGGTACCATGGTCTAAGCGACTGCTACTGCGTGGCTTTGAGGC 3′
hemSantisensavl	Antisens *hemS*	*B. henselae*	5′ CCCTCTAGAATGTCATATACAGCCGAAAT 3′
hemSantisensamt	Antisens*hemS*	*B. henselae*	5′ CCCGGATCCCTAAGCGACTGCTACTGCGTG3′
AmtAmtyfeX	Upstream of *yfeX*	XL1-blue	5′ ATTGTGGCGTTAATCTGGCTGCTG3′
AmtAvlyfeX	Upstream of *yfeX*	XL1-blue	5′GCGGATGAATGGCAGAAATTTGTTCCTCCTGAAAATAATAATGC3′
Cat5	Cmp cartridge	MG1655 *ttdA*::Cmp	5′ AATTTCTGCCATTCATCCGC3′
Cat3	Cmp cartridge	MG1655 *ttdA*::Cmp	5′ TTGATCGGCACGTAAGAGGT3′
AvlAmtyfeX	Downstream of *yfeX*	XL1-blue	5′ACCTCTTACGTGCCGATCAATTACTTCTGCTTTAACGCCGCATAC3′
AvlAvlyfeX	Downstream of *yfeX*	XL1-blue	5′ TTTAAACCGCAACAAATTGCCGCC3′

### Constructions of the vector for decreasing HemS amount in *B. henselae*

The entire coding region of *hemS* was amplified by PCR from the *B. henselae* chromosomal DNA using primers hemSantisensamt, containing a BamHI restriction site, and hemSantisensavl, containing an XbaI restriction site (Table 2). The 1054 bp PCR product was purified, digested with BamHI and XbaI and then ligated with plasmids pNS2Trc digested with BamHI and XbaI to give pNS2Trc*::hemS*_AS_. Ligation mixtures were introduced in *E. coli* strain XL1 blue using calcium chloride method. Transformants were screened, using PCR method with hemSantisensamt, and hemSantisensavl primers. Six PCR positive clones were then sequenced.

### Expression and Purification of HemS His-tagged protein

Strain JP313 (pBAD24::*hemS*_his_) was grown overnight at 37°C in LB medium containing 50 µg/ml Ampicillin. One liter of LB medium, containing 50 µg/ml Ampicillin, was inoculated at OD600 of 0.05 with the overnight culture. Bacteria were grown at 37°C to an OD600 of about 0.5. Arabinose at 0.2% final concentration was added and bacteria grown for an additional 4 h at 37°C. Bacteria were harvested by centrifugation for 10 min at 3000 g at 4°C, and the pellet was suspended in 20 ml binding buffer (50 mM Tris-HCl pH 8.0, 2.5 mM MgSO_4_, 10 mM imidazole, 0.05%Triton). Lysis of bacteria was obtained by sonication (7 s sonication followed by a 3 s pause) during 30 min. The suspension was then centrifuged at 13,000 g for 30 min at 4°C. The supernatant, containing the soluble fraction was mixed with 500 µl of Ni-agarose beads (Qiagen), previously pre-equilibrated with the binding buffer, and the mixture was incubated 1 hour with gentle shaking at 4°C and purified following the manufacturer's protocol. Purified protein was dialyzed twice against a buffer containing 50 mM Tris-HCl to eliminate any residual imidazole. The protein was estimated to be >95% pure through SDS gel electrophoresis and was stable for several months when kept at −20°C with 20% glycerol.

### Heme binding assay

After migration in SDS-PAGE, the heme binding ability of HemS was investigated according to the protocol of Vargas [32]. Briefly, samples were mixed with loading buffer in which no DTT was added. And samples were not boiled before electrophoresis. 4 µg of HemS protein and 5 µg of BSA were separated on 12% SDS-PAGE. One gel was stained with coomassie brilliant blue R. Another gel was transferred to nitrocellulose by the general methods of Towbin *et al.* [33]. Heme-binding blot was done according the protocol of Carroll *et al.* [34]. Briefly, the resulting blots were rinsed with TBST buffer (10 mM Tris-HCl pH 8.0, 150 mM NaCl, 0.1% Tween 20) three times for 10 min and subsequently probed for 1.5 h with TBS containing heme (10^−6^ M) at room temperature. The nitrocellulose was washed three times for 10 min with TBST at room temperature. Heme was visualized by it intrinsic peroxidase activity using enhanced chemiluminescence (ECL) reagents (Amersham pharmacia, Piscataway, N.J). Heme binding protein bands were visualized by exposing the blot to autoradiographic film (Labscientific, Livingston, N.J.).

### Absorption spectroscopy

Heme binding assays also were carried out using absorption spectroscopy method. HemS and heme were respectively diluted to 10 µM, and 200 µM in Tris-HCl 50 mM pH 8.0. Aliquots of heme (raising heme concentration from 1 µM to 20 µM final concentration) were successively added into the cuvette containing 100 µl of 10 µM HemS. Absorbance spectra from 300 nm to 700 nm were recorded 5 mins after each heme addition on a nanodrop 2000 spectrophotometer. Experiments were performed in triplicate.

### Reaction of HemS-heme complex with NADPH-cytochrome P450 reductase

The reaction was performed according to Zhu *et al* [35] and Skaar *et al* [19]. Human cytochrome P450 oxidoreductase (Sigma–Aldrich) was added to the HemS-heme complex (10 µM) at a ratio of reductase to HemS equal to 0.3∶1 in a final volume of 100 µl 50 mM Tris-HCl (pH 8.0). Initiation of the reaction was carried out by the addition of NADPH in 10 µM increments to a final concentration of 100 µM. The spectral changes between 300 and 700 nm were monitored after each addition. Experiments were performed in triplicate.

### Reaction of HemS-heme complex with ascorbate

Ascorbic acid-dependent degradation of heme was monitored spectrophotometrically as previously described [35]. HemS-heme complex (10 µM) in 50 mM Tris-HCl (pH 8.0) was incubated with ascorbic acid (10 mM), and the spectral changes between 300 and 700 nm were recorded every 1 min. Experiments were performed in triplicate.

### Antibodies preparation

200 µl of an emulsion containing purified HemS (10 µg), ISA 61 VG adjuvant (Seppic Paris France) (120 µl) and completed with NaCl 0.9% were inoculated twice (with one month interval) by subcutaneous route in C57B6 mice (Charles River) (Ethic committee Anses/ENVA/UPEC agreement n°:14/06/2011-1). Two weeks after the second inoculation, 200 µl blood samples were collected each 3 week, using retro orbital bleeding method. Blood samples were centrifuged twice (3600 rpm 5 min) and sera were stored at −20°C. Before use, unspecific antibodies were removed by incubating the immune serum with *E. coli* cell extract 1 hour at 4°C and centrifugation 10 min at 8000 rpm. The supernatant was then used as serum.

### Protein analysis by Electrophoresis

Proteins were analyzed by 12% sodium dodecyl sulfate-polyacrylamide gel (SDS-PAGE) electrophoresis [36], followed by Coomassie blue staining.

### Immunoblot analysis

Sodium dodecyl sulfate-polyacrylamide gel electrophoresis (SDS-PAGE) and immunoblotting for detecting the decrease expression of HemS in *B. henselae* were performed as follows: *B. henselae* (pNS2Trc) and *B. henselae* (pNS2Trc*::hemS*_AS_) were harvested after 5 days of growth on CBA plates. Proteins contained in 20 µg of each sample were separated by 12% SDS-PAGE and transferred to a nitrocellulose membrane (Hybond-C Extra, GE Healthcare) according to Towbin *et al* [33]. Nonspecific binding sites were blocked with 5% skim milk in TBS-Tween 20(0.05%). The immunoblot was probed with polyclonal mice sera raised against recombinant HemS (1∶200), followed by a 1∶1,000 dilution of a rabbit anti-mice IgG alkaline phosphatase-conjugated secondary antibody (Sigma ref. A4312). The binding of antibodies to HemS was revealed using chemiluminescence reagents BCIP/NBT solution following the manufacturer's instructions (Sigma).

### Protein assay

The concentration of the protein was determined by BC Assay protein Quantitation kit (interchim)

### Statistical analysis

Data was expressed by mean ± standard errors of the means. The statistical analysis was performed using GraphPad Prism 5 software for Windows. Statistical significance of the data was ascertained by use of the Student's *t* test. A value of P<0.05 was considered significant.

## Results

### HemS from *B. henselae* is able to complement *E. coli* mutants impaired in iron release from heme

**Figure 1:**
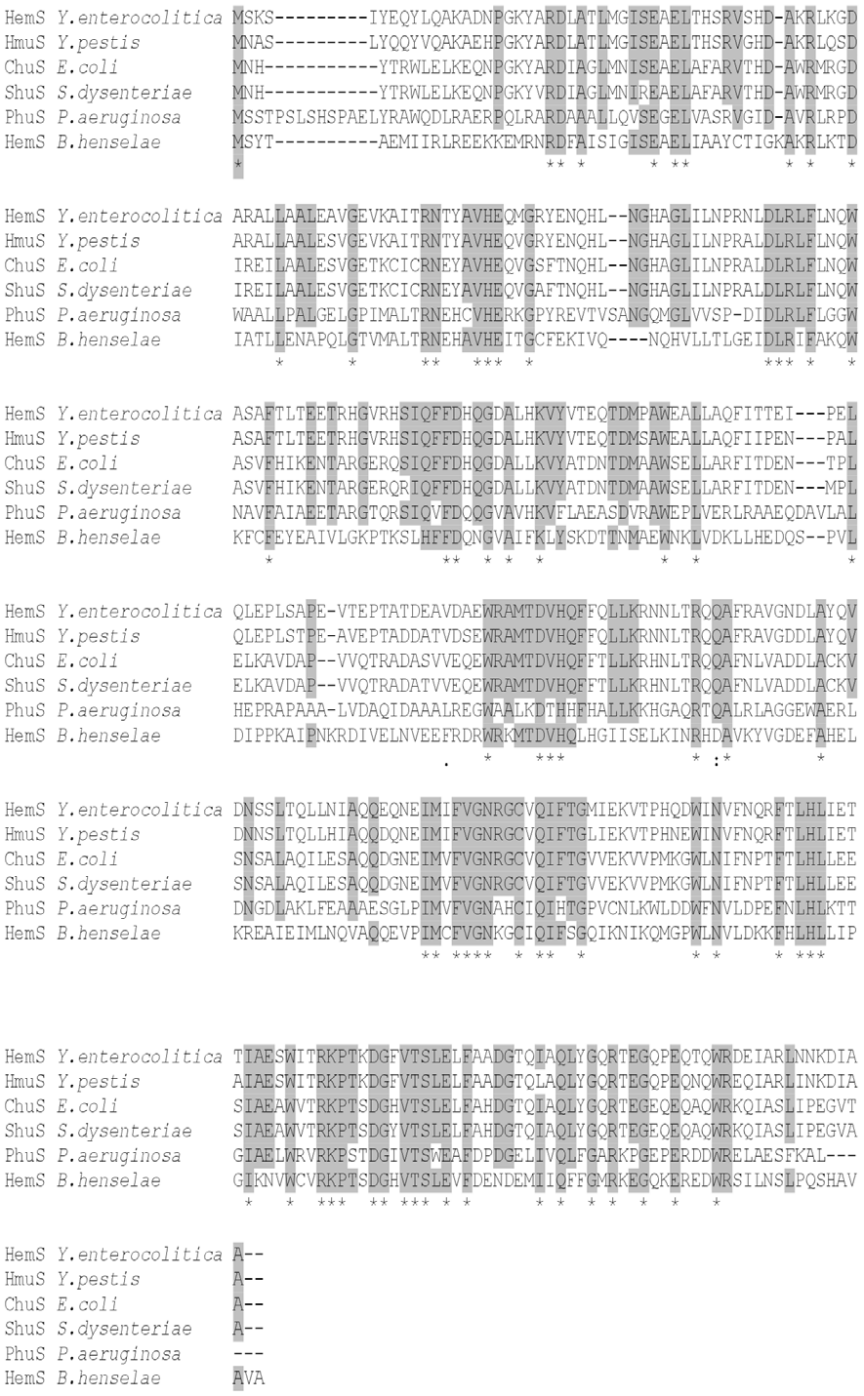
HemS and heme degrading or trafficking enzymes sequence alignment. ClustalW alignment of HemS from *B. henselae*, PhuS *from Pseudomonas aeruginosa* [52], ChuS from *E. coli* [46], ShuS from *Shigella dysenteriae* [43], HemS from *Yersinia enterocolitica* [21], HmuS from *Yersinia pestis* [47]. This alignment was generated by Clustal W. Amino acids conserved in five or more polypeptides are highlighted in grey. Amino acids conserved in all protein are indicated with a star.

**Figure 2:**
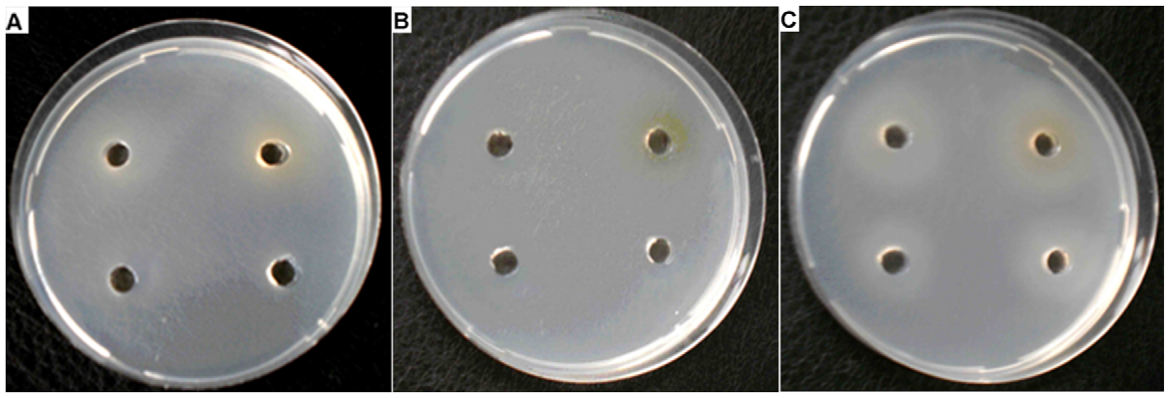
Functional complementation of the *E. coli* mutants impaired in iron release from heme. *E. coli* strains FB8.27 pAM*::hasR* (pBAD24) (**A**), FB8.27 *efeB::Kan yfeX::Cmp* (pAM*::hasR*) (pBAD24) (B) and FB8.27 *efeB::Kan yfeX::Cmp* (pAM*::hasR* ), (pBAD24::*hemS*_his_) (C) were tested for the use of heme as an iron source on iron depleted medium M63 (Gly 0.4%, Ara 0.2%, Dip 70 µM, Spc, Amp). Growth around the wells containing 1 µM, 5 µM, 10 µM, or 50 µM Hb were performed as described in “Materials and Methods”. Growth around the wells was assessed by visible turbidity in the agar. These pictures were taken after 48 hours of growth at 37°C. Experiment was repeated three times. A representative result is presented.

*Bartonella* genomes contain a gene encoding for HemS or HmuS that could likely be involved in the release of iron from heme. These proteins, which are 341 AA to 347 AA in size, share 72% to 77% identity. Searching for homologues of these proteins in bacterial genomes showed that these proteins share homology with numerous polypeptides annotated as heme degrading or heme trafficking enzymes. When searching for homologies with functionally characterized heme degrading enzymes and heme trafficking enzymes, HemS/HmuS from *Bartonella* shared 35% to 42% identity with some of these polypeptides (Fig. 1). Analysis of the identical regions did not allow predicting that HemS/HmuS from *Bartonella* were heme degrading enzyme or heme trafficking proteins. To attempt defining the HemS activity from *B. henselae*, its structural gene was amplified using a forward primer designed to add a 6×His-tag at its N-terminus, and cloned in plasmid pBAD24. The recombinant plasmid pBAD24::*hemS*_his_ was introduced in *E. coli* strain FB8.27 *efeB::Km yfeX::Cmp* (pAM*::hasR*) to check for complementation ability. This *E. coli* strain, similarly with strain FB8.27 (pAM*::hasR*), which cannot grown on an iron depleted medium since it is impaired in enterobactin biosynthesis [37]. When heme is added on iron depleted medium, strain FB8.27 (pAM*::hasR*) can grow, due to the presence of the HasR heme transporter from *Serratia marcescens* contained in plasmid pAM*::hasR* [38]. Heme is transported through the outer membrane by HasR, and the deferrochelation activity of EfeB and YfeX allows the release of iron required for growth [24]. In *E. coli* train FB8.27 *efeB::Km yfeX::Cmp* (pAM*::hasR*), deferrochelation activity is absent and consequently, heme dependent growth was abolished. When *hemS* from *B. henseleae*, was expressed in strain FB8.27 *efeB::Km yfeX::Cmp* (pAM*::hasR*) (pBAD24::*hemS*_his_) it restored the heme dependent growth on iron depleted medium (Fig. 2). This result clearly indicates that HemS activity allows the release of iron from heme *in vivo*.

### Expression and Purification of HemS His-tagged protein

To produce and purify the recombinant HemS protein from *E. coli*, plasmid pBAD24::*hemS*_his_ was introduced into strain JP313. To check for amounts of HemS in *E. coli* strain JP313, SDS gel electrophoresis (PAGE) was used to compare protein extracts of the strain JP313 (pBAD24::*hemS*_his_) and JP313 (pBAD24). A supplementary visible band of 40 KDa was observed only for the strain containing the recombinant plasmid pBAD24::*hemS*_his_ grown in the presence of arabinose (data not shown). In strain JP313 (pBAD24::*hemS*_his_), grown in the presence of 0.2% arabinose, His-tagged HemS was expressed as a soluble protein. The protein was purified by Ni-agarose affinity chromatography, yielding a distinct protein (95% pure) migrating at ∼40 KDa as a distinct band on SDS-PAGE (Fig. S1). This size was found to be in accordance with the predicted size.

### HemS can bind heme specifically *in vitro*

**Figure 3:**
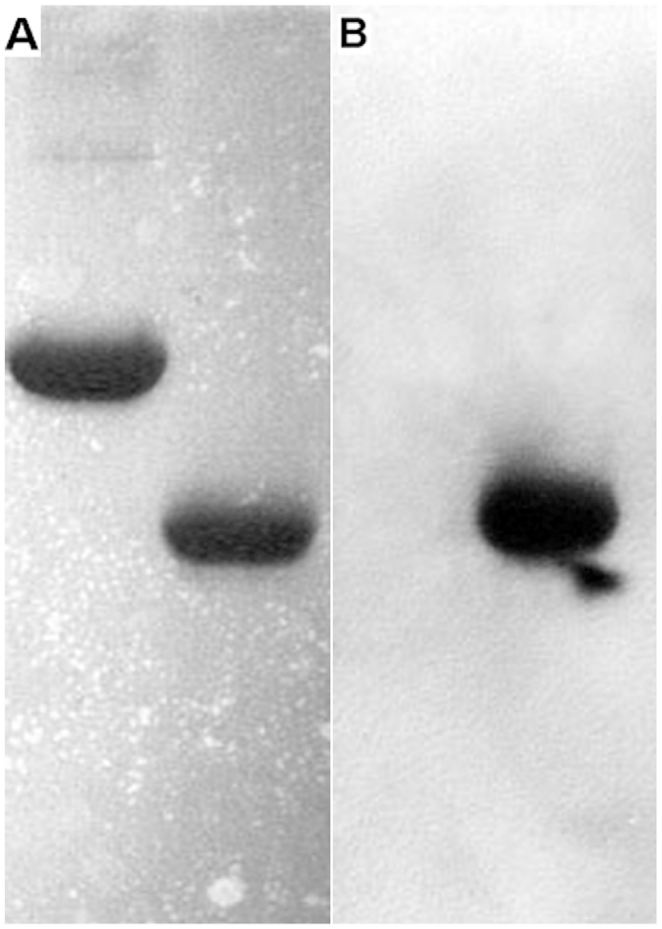
HemS heme blotting. After SDS gel electrophoresis, one gel was stained with comassie brilliant blue R. Another gel was transferred to a nitrocellulose filter to do heme blotting and detected by ECL. (A) Coomassie blue staining: Line 1, 5 µg BSA; Line 2, 4 µg HemS; (B) Heme binding: Line 1, 5 µg BSA; Line 2, 4 µg HemS. Experiment was performed in triplicate and a single representative experiment is presented.

To test if pure HemS can bind heme specifically *in vitro*, a standard method, which has already used for detected heme binding of cytochrome C, was used. Pure HemS (4 µg) and BSA (5 µg) were run in two SDS-PAGE gels. One gel was stained with coomassie brilliant blue R (Fig. 3A). Another gel was transferred to a nitrocellulose filter to perform heme blotting and subsequent detection by ECL method. Pure HemS was able to bind heme added at 10^−6^ M concentration (Fig. 3B). In contrast, in our condition assay, BSA was unable to bind heme (Fig. 3B). This latter result underlines the specificity of the HemS heme binding. Also, a crude extract obtained from *E. coli* strain JP313 (pBAD24::*hemS*_his_), expressed a protein of about 40 KDa that can bind heme. In contrast such a protein was not observed in a crude extract obtained from strain JP313 pBAD24 (data not shown). Taken together, these above data demonstrate that HemS from *B. henselae* is able to bind heme *in vitro*.

### HemS binds heme with a 1/1 stoechiometry

**Figure 4:**
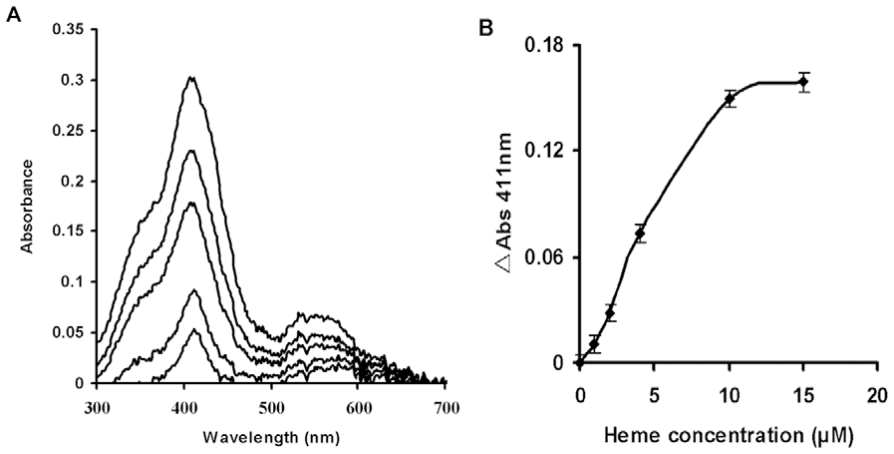
Binding of heme to HemS. (A): Increasing amounts of heme (1 µM–20 µM final concentration) were added to HemS (10 µM) as decribed in “Materials and Methods” and the spectrum (300 nm–700 nm) was recorded after 5 min for each addition. The Soret band at 411 nm increases with each addition of heme as demonstrated by absorbance peak increases at 411 nm. (B): Absorbance at 411 nm was measured for each sample and plotted versus heme concentration. Experiments were performed in triplicate and a single representative experiment is presented.

The binding of heme by HemS was also assessed spetrophotometrically. The spectral properties of heme changed when bound to a protein giving a specific Soret band. The spectrum of HemS-heme complex showed a peak at 411 nm (Fig. 4A). Titration of 10 µM HemS solution with increasing amount of heme was used to check for the HemS heme binding properties (Fig. 4B). The absorption at 411 nm increase leveled off at about 10 µM heme, showing a 1∶1 stoichiometry of heme to HemS (Fig. 4B).

### HemS can degrade heme *in vitro*

**Figure 5:**
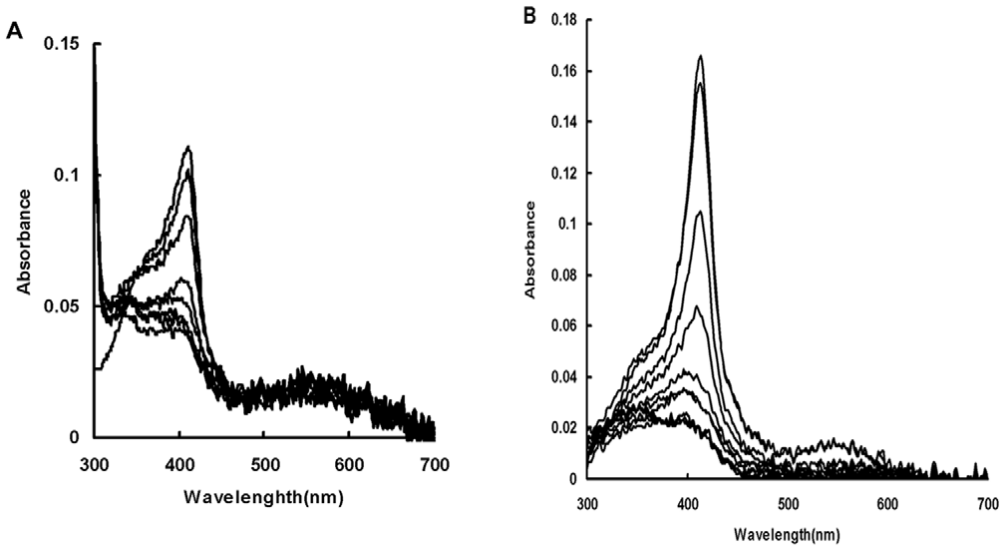
HemS dependent degradation of heme. (A): 10 mM final concentration of ascorbate was added to the HemS-heme complex (10 µM). The spectral changes from 300–700 nm were recorded every 1 min. (B): Cytochrome P450 reductase was added to 10 µM HemS-heme complex with a 0.3∶1 molar ratio and heme degradation was initiated by adding NADPH 10 µM increments to a final concentration of 100 µM. The spectra were recorded from 300–700 nm after each addition. All Experiments were performed in triplicate and a single representative experiment is presented.

Various monoheme-protein complexes like heme oxygenases [39], and other biochemically uncharacterized heme degrading enzymes [40], [20] were shown to degrade heme *in vitro* in the presence of electron donors like, ascorbate or Cytochrome NADPH-cytochrome P450 reductase. We thus tested whether HemS was able to degrade heme *in vitro* first in the presence of ascorbate or Cytochrome NADPH-cytochrome P450 reductase. The HemS-heme complex was incubated with ascorbic acid (5 mM), and the spectral changes between 300 and 700 nm were recorded every 1 min. As shown in Fig. 5A, the disappearance of the Soret band was nearly complete 5 min after addition of ascorbate. In the absence of ascorbate, the Soret band was stable at least for more than 30 min (data not shown). In a second experiment, HemS dependent heme degradation was measured spectrophotometrically using human cytochrome P450-NADPH as the electron donor. Cytochrome P450 reductase was added to HemS-heme complex and heme degradation was initiated by adding NADPH and the spectral changes between 300 and 700 nm spectral were recorded. The Soret band decreased after each addition of NADPH, and disappears after addition of 100 µM NADPH (Fig. 5B). The Soret band of HemS-heme complex did not change if NADPH or cytochrome P450 were added alone into the mixture (data not shown). And heme degradation did not occur in the mixture containing only cytochrome P450-heme-NADPH (data not shown). All these data demonstrate that HemS is able to degrade heme *in vitro* through an enzymatic dependent process that requires addition of electron donors.

### The *hemS* knockdown increased *B. henselae* sensitivity to hydrogen peroxide

**Figure 6:**
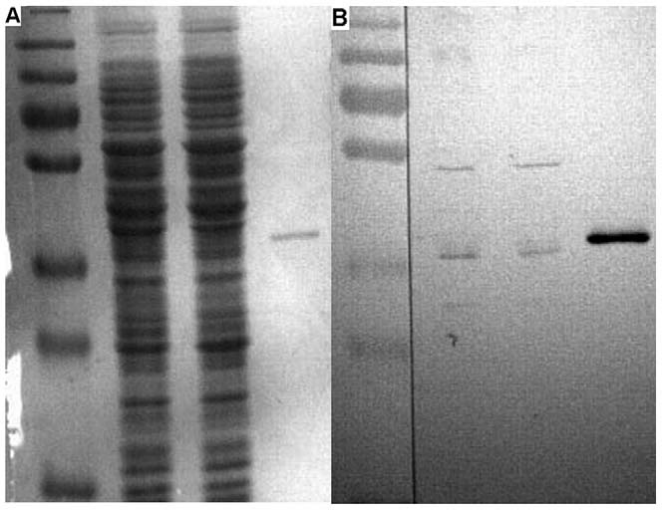
Detection of HemS expression level in *B. henselae* pNS2Trc and *B. henselae* pNS2Trc::hemS_AS_ by immunoblotting. 20 µg samples of *B. henselae* pNS2Trc (1) and *B. henselae* pNS2Trc::*hemS*_AS_ (2), 20 ng sample of purified his-tagged HemS (3) were loaded on SDS-PAGE. After electrophoresis, one gel was stained with comassie brilliant blue R (A). Another gel was transferred to a nitrocellulose filter to do immune blotting as decribed in “Materials and Methods” (B). Measurement of HemS band intensity using Image J software gave the following results: *B. henselae* pNS2Trc: mean gray value: 24, integrated density: 3218; *B. henselae* pNS2Trc*::hemS* AS: mean gray value: 14, integrated density: 2075.

As seen above, when expressed in *E. coli*, HemS was able to provoke the release of iron from heme. Biochemical results strengthen this observation. The question arises whether HemS activity is required for *Bartonella*. According to the conclusions provided by analysis of the genome content of *Bartonella*, we hypothesized that HemS is required for growth since its activity provides iron source when bacteria are grown in aerobic condition. Preliminary unsuccessful assays to disrupt *hemS* strengthened this hypothesis (data not shown). Lacking appropriate genetic tools, it is presently not possible to generate mutant for genes presumed to be essential for *B. henselae*. However it is possible to analyse the effect of the decrease of an essential gene product in this genus via knocking down gene expression. This method was used successfully to investigate the function of genes in *B. henselae* [41], [42]. We cloned *hemS* of *B. henselae* into a vector that allowed high level expression, pNS2Trc [41]. The gene was oriented in the reverse orientation such that the antisense strand was transcribed. Plasmids pNS2Trc and pNS2Trc*::hemS_AS_* were introduced into *B. henselae* by electroporation. We first checked if the level of HemS was decreased in strain *B. henselae* (pNS2Trc*::hemS_AS_*) as compared with strain *B. henselae* (pNS2Trc) using western blot experiment performed with anti HemS mice antibodies. As seen in Fig. 6B, the level of HemS contained in strain *B. henselae* (pNS2Trc*::hemS_AS_*) was lower than in strain *B. henselae* (pNS2Trc). Strains *B. henselae* (pNS2Trc) and *B. henselae* (pNS2Trc*::hemS_AS_*) were then tested for growth on both on CBA plates and in Schneider's medium. Our results showed that the knock down of HemS in strain *B. henselae* (pNS2Trc*::hemS_AS_*) did not significantly decrease its growth ability. Thus, the level of HemS in *B. henselae* (pNS2Trc*::hemS_AS_*) is sufficient to support a normal growth on CBA plates and in Schneider's medium.

**Figure 7:**
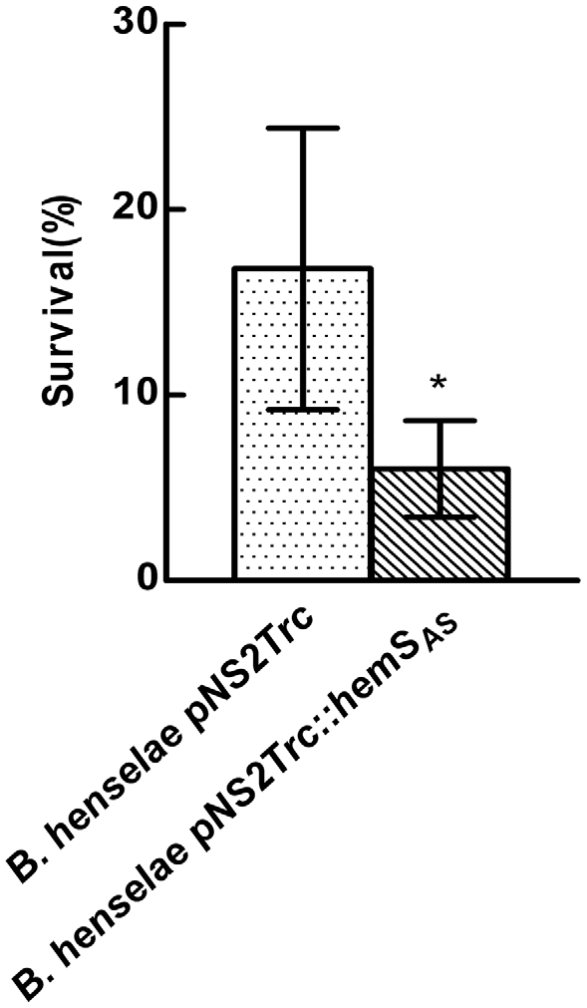
HemS knockdown decreases *B. henselae* ability to face exposure to H_2_O_2_. *B. henselae* pNS2Trc and *B. henselae* pNS2Trc::*hemS*_AS_ were challenged with 10 mM H_2_O_2_ as described in “Materials and Methods”. Experiments were performed in triplicate and a single representative experiment is presented.

HemS can degrade heme and thus can prevent its accumulation in the cytoplasm. Consequently, HemS could prevent the deleterious effects of heme accumulation in the cytoplasm. Therefore a decrease of HemS level in the cytoplasm could induce heme accumulation and lead to a higher sensitivity to oxidative stress. Such protecting activity against heme toxicity was demonstrated for ShuS that was evidenced to promote heme utilization in *Shigella dysenteriae*. In this bacteria, *shuS* disruption did not increased sensitivity to hydrogen peroxide [43]. *Shigella dysenteriae* genome (http://www.ncbi.nlm.nih.gov/genome/?term=Shigella%20dysenteriae%20) contains genes endoding for KatG, AhpC, and AhpF Alkyl Hydroperoxide Reductase, that are involved in hydrogen peroxide degradation [44]. Also, OxyR that regulates the response to H_2_O_2_ induced oxidative stress is present [44]. Analysis of the *Bartonella* genomes indicated that these bacteria would not be able to face oxidative stress using canonical pathways since many genes involved in oxidative stress response are not present. Homologs of genes encoding for hydrogen peroxide degrading enzymes like KatG and KatE catalases, AhpC, and AhpF Alkyl Hydroperoxide Reductase, Dps and OxyR are absent in *Bartonella* genomes (http://www.ncbi.nlm.nih.gov/protein?term=zwf%20Bartonella%20henselae). Nevertheless, a previous report showed that *Bartonella bacilliformis* was able to sustain successfully a 30 minutes exposure to 1 mM H_2_O_2_ [45]. This latter result strongly suggested that cellular activities allow this bacterium to face oxidative stress generated by exposure to H_2_O_2_. HemS, that is involved in heme disruption, could be an actor of this defense against oxidative stress. Therefore, we tested the effect of *hemS* knock down on the ability of *B. henselae* to face a 30 minutes exposure to 1 mM and 10 mM hydrogen peroxide. After exposure to 1 mM H_2_O_2_, the survival was about 50% for both strains. After exposure to 10 mM H_2_O_2_, the survival was about 20% for the control strain *B. henselae* (pNS2Trc). With strain *B. henselae* (pNS2Trc::*hemS_AS_*), the survival was decreased by three fold (Fig. 7). This result showed that lowering HemS level in *B. henselae* enhanced its sensitivity to H_2_O_2_.

## Discussion

In this report, we investigated the function of HemS in *B. henselae*. Homologs of this protein are present in all the *Bartonella* genome that have been sequenced. This underlines the importance of its function for these *Alphaproteobacteria*. Predicting an important role for HemS in *Bartonella* is mainly driven by the fact that these bacteria use heme as iron source. The use of heme as iron source requires its transportation through the outer membrane, the periplasm, and the inner membrane. The release of iron from heme inside the cytoplasm requires an enzymatic activity [15]. An iron release activity from heme was demonstrated in many bacteria, using physiological tests. Some of these activities were also biochemically demonstrated and characterized. In most cases, the release of heme was the consequence of the protoporphyrin ring degradation [15]. Heme oxygenase first characterized in *Corynebacterium diphteriae* [39], releases heme by degrading heme to forming α-billiverdin, CO and free iron. Others heme oxygenases that degrade heme and release iron in the presence of a reducing agent were characterized first, in *Bacillus anthracis* [19]. Members of these two classes of heme oxygenases were identified in various Gram negative and Gram positive bacteria [15]. Finally, a deferrochelation activity was shown to be responsible for the release iron from heme in *E. coli*. In this latter case, the release of iron occurs without breakage of the tetrapyrrol skeleton [24]. For some other bacteria, like *Yersinia enterocolitica*, the heme degrading activity was demonstrated, but the reaction products were not characterized. This was the case for HemS from *Yersinia enterocolitica* [21], ChuS from *E. coli* O157:H7 [46]. Other proteins involved in the use of heme, and sharing significant sequence homology with heme degrading enzymes, were characterized in *Yersinia pestis* [47] or in *Shigella disenteriae* [43]. For HemS from *B. henselae*, both complementation abilities and biochemical assays show that HemS can degrade heme. Similarly with IsdG from *Bacillus anthracis*, purified HemS from *B. henselae* can degrade heme in the presence of a reducting agent. During the heme degradation in the presence of NADPH-cytochrome P450 reductase, the soret band of the heme-HemS complex only decreased in intensity. This was not the case for HmuO from *Corynebacterium diphtheriae* since the Soret band wavelength of the heme-HmuO complex also vary during the heme degradation in the presence of NADPH-cytochrome P450 reductase [39]. Heme degradation enzymes of the IsdG family were characterized in *Alphaproteobacteria* like *Bradyrhizobium japonicum* [20] and *Brucella melitentis* [20]. The heme degradation in *Bradyrhizobium japonicum* produces billiverdin [20]. In *Brucella melitentis* the product of heme degradion by BmeII0706 was not characterized [20]. Similarly with BmeII0706 from *Brucella melitentis* [20], HemS from *B. henselae* and *B. birtlesii* (data not shown) exhibits a Soret band at 411 nm in the presence of heme, thus suggesting a similar environment for heme. Our study however does not show the products, which would be useful to distinguish true HO activity from peroxide-coupled oxidation. HemS knock down only provoked a slight slowdown growing effect when bacteria were grown on blood plates. When bacteria were grown in Schneider medium, no growing effect was observed.

The more striking effect related to HemS knockdown was to decrease the ability *B. henselae* to sustain exposure to hydrogen peroxide. This result suggests that HemS could be an actor of the pathway used by *Bartonella* to face oxidative stress. Since classical pathways used to face oxidative stress characterized in *E. coli* are not present in *Bartonella*, we hypothesized that *Bartonella* develop alternatives strategies to face oxidative stress encountered in erythrocytes or macrophages and during vectorisation by the cat flea *Ctenocephalides felis*. Inside erythrocytes, and in the flea gut, Hb and heme concentration are high [48]. Thus, HemS may be proposed to have a dual role for *Bartonella*, since its heme degrading activity allows iron supplying and the control of heme homeostasis.

## Supporting Information

Figure S1 **Purification if His-tagged HemS.** Purification of the His-tagged HemS protein was achieved by Ni-agarose purification followed by gel filtration. Purified protein (5 µg) was run on 12% sodium SDS-PAGE and stained with Coomassie blue. Line MW: molecular weight markers. Line 1: purified HemS.
(TIF)
