# Supplementary figures and images for: Heme Degrading Protein HemS Is Involved in Oxidative Stress Response of Bartonella henselae
Source: PLoS One. 2012 May 31;7(5):e37630. doi: 10.1371/journal.pone.0037630 (PMC3365110; doi:10.1371/journal.pone.0037630)

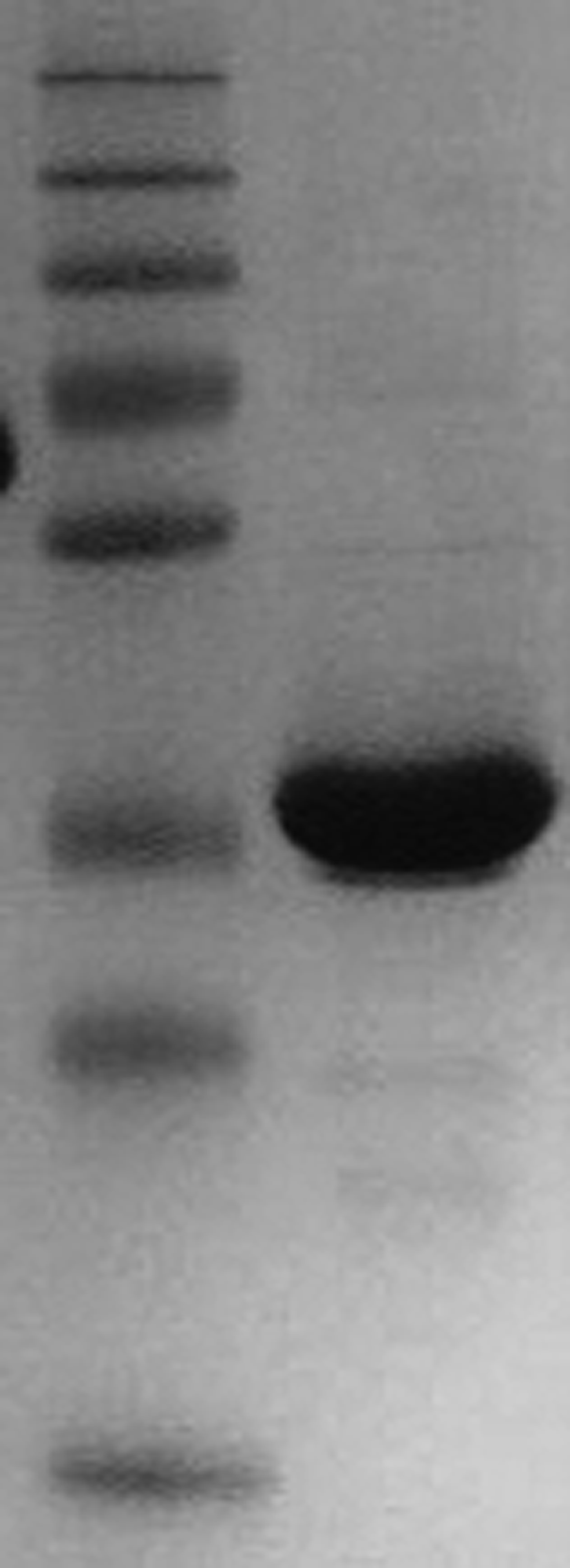

Supplement: Figure S1 — Purification if His-tagged HemS. Purification of the His-tagged HemS protein was achieved by Ni-agarose purification followed by gel filtration. Purified protein (5 µg) was run on 12% sodium SDS-PAGE and stained with Coomassie blue. Line MW: molecular weight markers. Line 1: purified HemS. (TIF) [file pone.0037630.s001.tif]
